# Compressing Deep Networks by Neuron Agglomerative Clustering

**DOI:** 10.3390/s20216033

**Published:** 2020-10-23

**Authors:** Li-Na Wang, Wenxue Liu, Xiang Liu, Guoqiang Zhong, Partha Pratim Roy, Junyu Dong, Kaizhu Huang

**Affiliations:** 1 Department of Computer Science and Technology, Ocean University of China, Qingdao 266100, China; alinagq@163.com (L.-N.W.); 15169829189@163.com (W.L.); liuxiang@stu.ouc.edu.cn (X.L.); gqzhong@ouc.edu.cn (G.Z.); 2 Innovation Center, Ocean University of China, Qingdao 266100, China; 3 Department of Computer Science and Engineering, Indian Institute of Technology Roorkee, Roorkee 247667, Uttarakhand, India; 2partharoy@gmail.com; 4 Department of Electrical and Electronical Engineering, Xi’an Jiaotong-Liverpool University, Suzhou 215123, China; kaizhu.huang@xjtlu.edu.cn

**Keywords:** deep learning, network compression, neurons, feature maps, agglomerative clustering

## Abstract

In recent years, deep learning models have achieved remarkable successes in various applications, such as pattern recognition, computer vision, and signal processing. However, high-performance deep architectures are often accompanied by a large storage space and long computational time, which make it difficult to fully exploit many deep neural networks (DNNs), especially in scenarios in which computing resources are limited. In this paper, to tackle this problem, we introduce a method for compressing the structure and parameters of DNNs based on neuron agglomerative clustering (NAC). Specifically, we utilize the agglomerative clustering algorithm to find similar neurons, while these similar neurons and the connections linked to them are then agglomerated together. Using NAC, the number of parameters and the storage space of DNNs are greatly reduced, without the support of an extra library or hardware. Extensive experiments demonstrate that NAC is very effective for the neuron agglomeration of both the fully connected and convolutional layers, which are common building blocks of DNNs, delivering similar or even higher network accuracy. Specifically, on the benchmark CIFAR-10 and CIFAR-100 datasets, using NAC to compress the parameters of the original VGGNet by 92.96% and 81.10%, respectively, the compact network obtained still outperforms the original networks.

## 1. Introduction

In order to solve challenging deep learning problems, such as pattern recognition and computer vision [1,2,3], researchers tend to design deep neural networks (DNNs) with complex structures and many neurons. However, as DNNs go deeper and deeper, the number of parameters increases dramatically. Therefore, huge storage space and long inference time are usually required by DNNs, which leads them to be only deployed on computational servers with graphics processing units (GPUs) [4]. Nevertheless, for more common mobile devices with limited storage and computing resources, network storage and running efficiency are very important factors. Therefore, it is almost impossible to directly apply large-scale DNNs on them. In contrast, shallow neural networks are much easier to store and more computationally efficient. However, shallow neural networks cannot generally match the performance of DNNs. To that end, it is necessary to compress DNNs and deploy them on devices with limited storage and computing resources.

In most DNNs, fully connected layers and convolutional layers are two widely used building blocks. In particular, the fully connected layers have dense connections and correspondingly a large number of parameters. Alternatively, the convolutional layers are important for learning layer-wise representations of the input data and are always computationally intensive. For example, in VGGNet-16, the parameters of the fully connected layers account for more than 90% of the total ones, whilst the floating-point operations (FLOPs) account for only less than 1% of the total FLOPs [5]. However, some existing network compression methods can only work on fully connected layers [6], while some approaches to compress convolutional layers require an additional sparse BLASlibrary or special hardware support [7,8,9].

In this paper, we introduce a systematic DNN compression method built on neuron agglomerative clustering (NAC), which is mainly applied to the neurons/feature maps of fully connected layers and convolutional layers. In particular, NAC agglomerates neurons/feature maps in the network instead of pruning individual weights at a time. For concreteness, we attain similar neurons/feature maps in the neural network through agglomerative clustering and then respectively agglomerate them and their related connections together. As NAC does not cause sparse connections, it does not require an additional library or hardware support. Last but not the least, during the process of agglomerative clustering, NAC does not need to use the original training data, but only the weights and biases connected to the neurons/feature maps.

The rest of this paper is organized as follows: In the following section, we briefly introduce some related work, including some neural network compression approaches and the agglomerative clustering algorithm used in the proposed network compression method. In Section 2, we describe the proposed method, NAC, in detail. The experimental results are reported in Section 3, while Section 4 concludes this paper with remarks.

## 2. Related Work

In this section, we first review some mainstream methods for network compression, including pruning, low-rank decomposition, compact convolutional filters’ design, knowledge distillation, and weight quantization. Next, we briefly introduce the agglomerative clustering algorithm used in the proposed network compression method.

### 2.1. Pruning

The main idea of pruning is to remove the relatively less informative and less important parameters in a trained neural network model, thereby reducing the amount of parameters in the network [10,11,12]. Reference [13] proposed a pruning method that regards the pruning problem as a combinatorial optimization problem: selecting an optimal combination from a number of weight parameters to minimize the value of the loss function. Moreover, some work for directly judging the importance of filters via the norm value of filters has been proposed. For example, in [5], the authors directly deleted the filters with a small $L_{1}$ norm in the current layer and then retrained the network to improve the accuracy of the pruned model. In [14], the authors considered that pruning approaches generally preserve two kinds of data for the compressed model, i.e., non-zero weights and meta-data, where meta-data are employed to help encode and decode the non-zero weights. Although it is possible to obtain an ideally small number of non-zero weights through pruning, existing sparse matrix coding methods still need a much larger amount of meta-data (which may be several times larger than non-zero weights), which will be a severe bottleneck for deploying very deep models. To tackle this problem, the authors proposed a layer-wise sparse coding (LSC) method to maximize the compression ratio by extremely reducing the amount of meta-data. LSC first divides a sparse matrix into multiple small blocks and removes zero blocks, then applies a signed relative index (SRI) algorithm to encode the remaining non-zero blocks (with much less meta-data). In [15], the authors found that a standard pruning technique naturally uncovers subnetworks whose initializations made them capable of effective training. Based on these findings, the authors articulated the lottery ticket hypothesis: dense, randomly-initialized, feed-forward networks contain subnetworks (winning tickets) that—when trained in isolation—reach test accuracy comparable to the original network in a similar number of iterations. The winning tickets the authors found have won the initialization lottery: their connections have initial weights that make training particularly effective. However, pruning strategies usually require many manual operations, e.g., judging the sensitivity of each layer, computing the norm of the filters, dividing the weight matrix, and identifying winning tickets, so that they need too much expertise and are not efficient.

In [16], Yu and Tian classified the pruning approaches into three categories according to the pruning granularity, i.e., single weight granularity, kernel weight granularity, and channel granularity. The optimal brain damage (OBD) method [17] belongs to the category of single weight granularity. It regards any weight parameter as a single parameter. It can improve the accuracy of prediction, yet cannot reduce the running time. At the same time, it takes too much time to prune the weights, so it only suits small networks. In order to improve the OBD method, Reference [18] introduced the optimal brain surgeon (OBS) method, which adds a step to update the weights based on recovery from surgery. OBS greatly improves OBD in accuracy and generalization ability. However, both of them need to update the significance of all parameters during iterative computation, which is time consuming. In [19], the authors successfully compressed the VGGNet-16 model, but their approach heavily depends on special software computing libraries and specialized hardware [20]. In [21], the concept of structured pruning was proposed, which fully uses the sparse regularization after pruning to speed up the network’s efficiency. The innovation of this method is that the authors put forward the concept of IntraKernelStridedsparsity, turning the fine-grained pruning into structured pruning. Channel granularity pruning methods do not rely on any sparse convolution calculation library and dedicated hardware. At the same time, they can obtain a high compression rate and greatly reduce the computing time of testing [5,22]. Reference [23] argued that most of the output neurons are zero after the activation function, which means that part of the basic network structure is redundant and not affected by the input data at all. Furthermore, the authors removed those units whose outputs are always zero for different inputs based on the statistical method and alternate retraining. Reference [13] combined the greedy pruning method and fine-tuning method of back propagation to ensure the generalization of the network after pruning. In particular, the authors proposed a method to approximately calculate the change of the loss function after removing some parameters based on the Taylor expansion. Reference [24] also put forward a kind of strategy for pruning the total neurons; the authors introduced a kind of maximizing output (Maxout) unit incorporating multiple neurons for more complex expression of the convex function and chose them based on each neuron’s response of partial correlation on the training set. However, these approaches determine the neurons or channels to be removed only based on defined rules. The similarity between neurons and feature maps cannot be automatically learned. Hence, to tackle this problem, we adopt agglomerative clustering algorithms to learn the similarity between neurons/feature maps and then merge them together.

### 2.2. Low-Rank Decomposition

In general, we represent the network weights in the form of tensors. Low-rank decomposition approaches decompose the original weight tensors into two or more smaller tensors [25,26,27]. Mathematically, either the four-dimensional convolutional kernel tensors or the two-dimensional fully connected layer weight matrices can be factorized using the low-rank decomposition method, thereby reducing the redundancy of the parameters. In [28], the authors decomposed the original convolutional kernels of size k×k into 1  ×k and k× 1 convolutional kernels using the low-rank decomposition technique. Similarly, Denton et al. first computed a low-rank approximation to the parameter tensors of the convolutional layers and then fine-tuned the network to restore its accuracy [25]. Furthermore, in [29], the authors performed the singular value decomposition of the parameter matrices and did not optimize the parameters using the stochastic gradient descent algorithm. In [26], an idea to explicitly model the output reconstruction error between the original and compressed CNNs was given, where the error is minimized to pursue a satisfactory rate-distortion after compression. In particular, a global error reconstruction method termed GERwas presented, which firstly leveraged an SVD-based low-rank approximation to coarsely compress the parameters in the fully connected layers in a layer-wise manner. Subsequently, such layer-wise initial compressions are jointly optimized in a global perspective via back-propagation. Based on dictionary learning, Reference [30] demonstrated that there is significant redundancy in the parameterization of several deep learning models. Given only a few weight values for each feature, it is possible to accurately predict the remaining values. Moreover, Reference [30] showed that not only can the parameter values be predicted, but many of them need not be learned at all. The authors trained several different architectures by learning only a small number of weights and predicting the rest. In the best case, the authors could predict more than 95% of the weights of a network without any drop in accuracy. However, since low-rank decomposition involves a computationally expensive decomposition operation, its implementation is not efficient in practical applications.

### 2.3. Compact Convolutional Filters Design

In traditional convolutional nets, the computation on the convolutional filters is generally dense. That is, all the parameters of each convolutional filter are non-zero, and each convolutional filter needs to be applied on all the feature maps. The compact convolutional filters reduce the storage and computational complexity of the convolutional nets via the specially designed convolutional filters. Chollet proposed the Xception network structure, which is based on the residual neural network [31] and replaces the convolution operation with the separable convolution [32]. The separable convolution first performs 1 × 1 convolution and then performs the channel-by-channel convolution. Zhang et al. proposed ShuffleNet, which uses point-wise group convolution and channel shuffling to construct a neural network model, thus reducing the computational complexity [33]. In general, the use of point-wise group convolution will prevent the information between different groups from circulating. The authors enabled the information flow through the channel shuffle operation, so as to obtain similar effects to the original convolution. However, the compact convolutional filters’ design can only be applied to the convolutional layers, and compared to the original model, the generalization of the convolutional nets with the compact convolutional filters’ design is arguably weaker.

More than designing compact convolutional filters, some work tries to construct compact convolutional networks directly. For the application of human activity recognition (HAR) using a smartphone, Reference [34] presented a fast and robust deep convolutional neural network structure, which only needs 0.0029 s to predict 12 complex activities in an online way with 95.27% accuracy. Additionally, Reference [35] proposed a convolutional structure: downsampling-convolutional restricted Boltzmann machines (D-CRBM) [36], and used it to replace the standard convolution to reduce parameters and computational consumption. Specifically, the variational autoencoder (VAE) model [37,38] composed of multiple D-CRBM layers was used to learn the hidden features of the sensing data. However, similar to the problems of compact convolutional filters’ design, the capability of compact convolutional networks may be limited due to their simple structures.

### 2.4. Knowledge Distillation

In 2014, Ba et al. proposed a method that can compress deep and relatively wide neural networks into shallow models. This shallow model can mimic the original models and achieve similar performance [39]. In 2015, Hinton et al. proposed the concept of knowledge distillation. By introducing soft targets encoded by the softmax function of the teacher network as a part of the whole loss function, knowledge distillation induces the training of the low-complexity student network and transfers knowledge from the teacher network to the student network [40]. Recently, some research tried to leverage knowledge distillation for compressing deep learning models [41,42,43,44,45,46]. Among others, Hanting et al. proposed a knowledge distillation method that does not require the participation of the original training data during model compression. By combining the ideas of the generative adversarial network (GAN) [47] and knowledge distillation, the student network is able to effectively learn the performance of the teacher network [48]. In [49], the authors proposed a knowledge distillation method based on the correlation between instances. Unlike the previous knowledge distillation method, the correlation congruence knowledge distillation (CCKD) method transfers not only the instance-level information, but also the correlation between instances. In a variety of tasks, CCKD obtains better performance than the original knowledge distillation method. However, knowledge distillation relies on the outputs of the softmax function, so that it is mainly suitable for classification applications.

### 2.5. Weight Quantization

DNNs usually have a large number of weight parameters. In order to reduce the storage space occupied by the parameters, weight quantization algorithms are widely used for deep neural network compression [19,50,51,52,53,54,55,56]. Basically, there are two categories of weight quantization algorithms. One is weight sharing, that is the weights of multiple connections in the network share the same one. For example, Cheng et al. proposed a simple yet effective cyclic projection method, which uses a cyclic matrix with a very small amount of storage to replace the original matrix, while using a fast Fourier transform to accelerate the calculation of the matrix [57]. The other class is low-precision quantization, which compresses the original network by reducing the number of bits required to represent each weight. For example, Ma et al. quantified the weights and activation outputs to eight bits and 10 bits, respectively [58], and Gysel et al. used fine-tuning to quantify the weights and activation outputs to eight bits [59]. In order to compress the network to a greater extent, approaches on binary representation of network parameters have been proposed. Specifically, Hubara et al. proposed the binarized neural network (BNN), by quantizing the weights and activation outputs to -1 or one, such that the neural network is greatly compressed [60]. However, when compressing deep networks with weight quantization algorithms, the classification accuracy may be greatly reduced.

### 2.6. Agglomerative Clustering Method

The agglomerative clustering method creates a hierarchical clustering tree by calculating the similarity between different categories of data points [61]. There are the bottom-up agglomerative method and top-down split method for creating a clustering tree. The agglomerative clustering refers to each sample point being initially regarded as a cluster, so that the size of the initial cluster is equal to the number of sample points, and then, these initial clusters are merged according to rules until a certain condition is reached or a certain number of categories is reached. The division clustering refers to initially classifying all samples into one cluster and then gradually dividing until a certain condition is reached or a given number of clusters is reached. Because in our proposed network compression method, we learn similar neurons in the same layer by neuron clustering algorithms and merge them together, agglomerative clustering is very suitable for the proposed method. The results obtained on the datasets used in the experimental section also demonstrate the superiority of the adopted agglomerative clustering algorithm.

## 3. The Proposed Approach

In this section, we first introduce the proposed network compression algorithm based on neuron agglomerative clustering (NAC) in detail. Then, we specify the application of NAC to the fully connected and convolutional layers.

### 3.1. Network Compression Based on Neuron Agglomerative Clustering

In general, neurons in the same layer of DNNs are redundant, which may require extra storage space and running time during learning. Here, we present a network compression algorithm based on neuron agglomerative clustering (NAC), which can be applied to both fully connected and convolutional layers and greatly reduce the redundancy of the neurons/feature maps.

In order to facilitate the following analysis, we first specify some notations. In a neural network, the activation output of a neuron is denoted as:

(1) $$a_{i}^{l}=f\left(\sum_{j=1}^{n_{l-1}}W_{i,j}^{l}a_{j}^{l-1}+b_{i}^{l}\right),$$

where *f* is the activation function, $n_{l-1}$ represents the number of neurons in the (*l* − 1)th layer, $W_{i,j}^{l}$ represents the weight between the *i*th neuron in the *l*th layer and the *j*th neuron in the (*l* − 1)th layer, $a_{j}^{l-1}$ is the the activation output of the *j*th neuron in the (*l* − 1)th layer, and $b_{i}^{l}$ is the bias of the *i*th neuron in the *l*th layer. According to Equation (1), the activation output of the *i*th neuron in the *l*th layer is uniquely determined by the weight $\left[W_{i,1}^{l},W_{i,2}^{l},W_{i,3}^{l},\cdots ,W_{i,n_{l-1}}^{l}\right]$, bias $b_{i}^{l}$, and the outputs of the previous layer. Therefore, each neuron can be expressed as:

(2) $$neu_{i}^{l}=\left[W_{i,1}^{l},W_{i,2}^{l},W_{i,3}^{l},\cdots ,W_{i,n_{l-1}}^{l},b_{i}^{l}\right],$$

where $neu_{i}^{l}$ represents the *i*th neuron in the *l*th layer.

For all neurons of the *l*th layer in the network, we write them together as a set:

(3) $$S_{l}=\left\{neu_{1}^{l},neu_{2}^{l},neu_{3}^{l},\cdots ,neu_{n_{l}}^{l}\right\}.$$

For each hidden layer *l* in DNNs, to find similar neurons, we perform the agglomerative clustering algorithm on the neuron set $S_{l}$ that includes all the neurons in the same layer. Concretely, in the beginning of the agglomerative clustering algorithm, we consider each neuron as a cluster and measure their similarity using the Euclidean distance. Then, the most similar pair of neurons is merged together, and their mean is taken as the new cluster center. Following that, we repeatedly agglomerate the clusters by minimizing the variances of them and update the cluster means, until the given compression ratio is reached [62]. To the end, we take into account the neurons in the same cluster that are similar to each other. Furthermore, the clustering center of each cluster is retained as a new neuron to represent the similar neurons in the corresponding cluster (i.e., multiple similar neurons in the same layer are agglomerated into a single cluster centroid). Specifically, in a network, neurons in layer *l* have connections with neurons in layer *l* − 1 and layer *l* + 1. After agglomerating multiple neurons in layer *l*, we need to adjust the architecture and weights to maintain the performance of the network. Therefore, we must retain the cluster assignment information of each neuron (i.e., into which cluster each neuron in the original network was specifically divided). This is very important for the subsequent architecture adjustment. We use $k_{l}$ to denote the number of cluster centers in the *l*th layer, and the cluster centroids set in the *l*th layer can be expressed as:

(4) $$P_{l}=\left\{p_{1}^{l},p_{2}^{l},p_{3}^{l},\cdots ,p_{k_{l}}^{l}\right\},$$

where $p_{i}^{l}$ represents the centroid of the *i*th cluster in the *l*th layer. The cluster assignment information of the *l*th layer is expressed as:

(5) $$R_{l}=\left[r_{1}^{l},r_{2}^{l},r_{3}^{l},\cdots ,r_{n_{l}}^{l}\right];$$

here, $r_{i}^{l}$ indicates into which cluster the *i*th neuron in the *l*th layer is divided. In the *l*th layer, neurons divided into the same cluster are agglomerated to the cluster center. To be more concrete, for the neuron $neu_{i}^{l}$, $r_{i}^{l}=m$ denotes that $neu_{i}^{l}$ is divided into the *m*th cluster, and then, $neu_{i}^{l}$ is replaced by the center of the cluster $p_{m}^{l}$. After neurons in a cluster are agglomerated, the connections between these neurons and related neurons must also be agglomerated accordingly. In other words, all related connections of the agglomerated neurons must be agglomerated to a new connection and connected to the clustering center $p_{m}^{l}$. Since neurons in the same cluster are similar, we use the addition operation to agglomerate connections. Here, we give a simple derivation process. First, we suppose that neurons in one cluster are the same. With this assumption, if $r_{i}^{l}=r_{j}^{l}$, then $neu_{i}^{l}=neu_{j}^{l}$ is satisfied. In particular, if the same input is fed into the network, these neurons will generate the same activation output. As a result, we can calculate the activation output of the *i*th neuron in the (*l* + 1)th layer as:

(6) $$\begin{array}{cc} a_{i}^{l+1} & =f(\sum_{j=1}^{n_{l}}W_{i,j}^{l+1}a_{j}^{l}+b_{i}^{l+1}) \end{array}$$

(7) $$\begin{array}{cc} \; & =f(\sum_{p=1}^{k_{l}}\left(\sum_{r_{j}=p}W_{i,j}^{l+1}a_{j}^{l}\right)+b_{i}^{l+1}) \end{array}$$

(8) $$\begin{array}{cc} \; & \approx f(\sum_{p=1}^{k_{l}}\left(\sum_{r_{j}=p}W_{i,j}^{l+1}{\hat{a}}_{p}^{l}\right)+b_{i}^{l+1}) \end{array}$$

(9) $$\begin{array}{cc} \; & =f(\sum_{p=1}^{k_{l}}{\tilde{W}}_{i,p}^{l+1}{\hat{a}}_{p}^{l}+b_{i}^{l+1}), \end{array}$$

where ${\tilde{W}}_{i,p}^{l+1}=\sum_{r_{j}=p}W_{i,j}^{l+1}$ means the agglomeration of the connections in the network and ${\hat{a}}_{p}^{l}$ represents the activation output of the *p*th cluster center in the *l*th layer. In the end, the connections corresponding to the similar neurons are also agglomerated together.

Informally, the proposed network compression method based on NAC is shown in Algorithm 1.
**Algorithm 1** Neuron agglomerative clustering.1:For each layer *l* in the network:2:Let $neu_{i}^{l}=\left[W_{i,1}^{l},W_{i,2}^{l},W_{i,3}^{l},\cdots ,W_{i,n_{l-1}}^{l},b_{i}^{l}\right]$;3:Construct neuron set $S_{l}=\left\{neu_{1}^{l},neu_{2}^{l},neu_{3}^{l},\cdots ,neu_{n_{l}}^{l}\right\}$;4:Cluster the neurons in set $S_{l}$ into $k_{l}$ groups using agglomerative clustering;5:Construct a set of cluster centroids $P_{l}=\left\{p_{1}^{l},p_{2}^{l},p_{3}^{l},\cdots ,p_{k_{l}}^{l}\right\}$;6:Agglomerate neurons of the same cluster into its cluster centroid;7:Remember the agglomerating list $R_{l}=\left[r_{1}^{l},r_{2}^{l},r_{3}^{l},\cdots ,r_{n_{l}}^{l}\right]$;8:Calculate ${\tilde{W}}_{i,p}^{l+1}=\sum_{j=1}^{n_{l}}I\left(r_{j}^{l}=p\right)W_{i,j}^{l+1}$, $i=1,\cdots ,n_{l+1}$, $p=1,\cdots ,k_{l}$, where I(·) is the indication function;9:Agglomerate connections of layer l+1 into ${\tilde{W}}_{i,p}^{l+1}$. Bias remains unchanged.

### 3.2. Applying NAC to Fully Connected Layers and Convolutional Layers

In this section, we specify how to apply the proposed NAC method to the fully connected layers and the convolutional layers.

**Figure 1:**
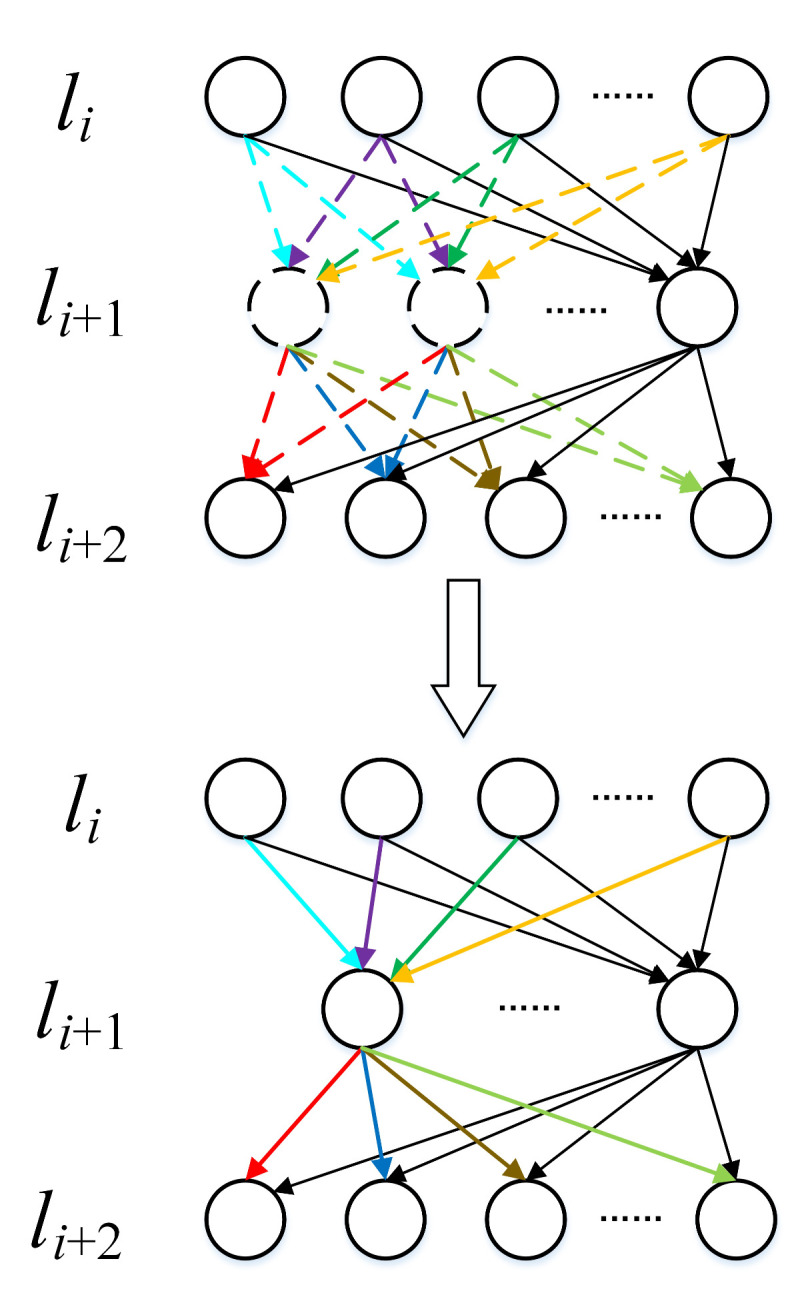
Agglomerating neurons in the fully connected layers.

The process of agglomerating neurons in fully connected layers is illustrated in Figure 1. The upper part of Figure 1 shows the structure of the neural network before agglomerating. The two neurons drawn with dashed lines in the $l_{i+1}$th layer are two similar neurons divided into the same cluster by the agglomerative clustering algorithm. The colored dashed lines indicate their connections with the neurons in the $l_{i}$th layer and the $l_{i+2}$th layer. After the two neurons and their related connections are agglomerated, we can obtain the compressed network structure as shown in the lower part of Figure 1. The two similar neurons in the $l_{i+1}$th layer are agglomerated into a cluster centroid obtained by the agglomerative clustering algorithm, and the connections between the neurons are also merged into new connections with the same colors, constructing a compact network structure.

For the application of NAC to the convolutional layers, we only need some small modifications to the convolutional filters. The rest of the processes are exactly the same as NAC applied to the fully connected layers. In the following, we introduce the application of NAC to the convolutional layers.

**Figure 2:**
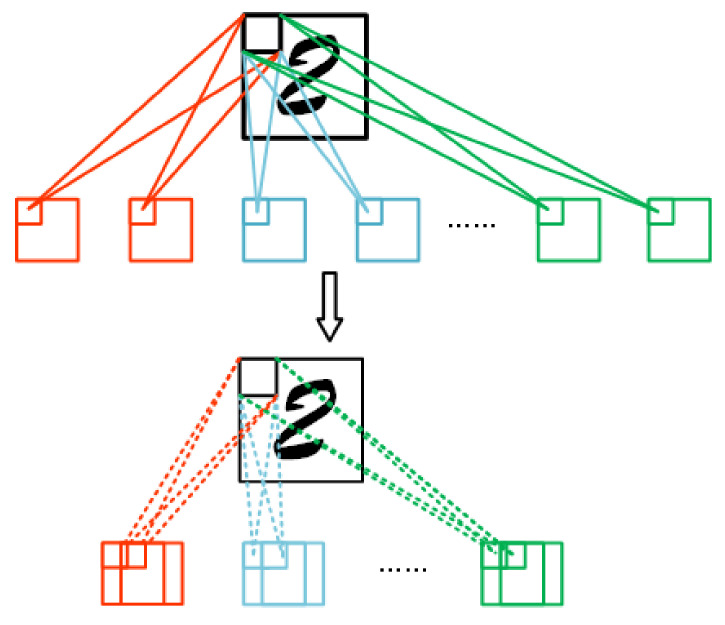
Agglomerating neurons in the convolutional layers.

In the convolutional layers, neurons in the same feature map share the same parameters (convolutional kernels and biases). These neurons also include the spatial information determined by their specific location. Therefore, we do not agglomerate these neurons directly to avoid destroying the effectiveness of the convolutional network. We consider agglomerating neurons from different feature maps. We represent the neurons in the same feature map as a tuple consisting of a convolutional kernel and bias. Before performing the agglomerating operation on the neurons, we first reshape this tuple {kernel,bias} into a one-dimensional vector, and then, the agglomerating operation is applied the same as for the fully connected layer. Finally, the agglomerated neurons are reshaped into the tuple composed of the convolutional kernel and bias, and the corresponding parameters are put back into the convolutional neural network to complete the agglomerating process and maintain the structure and performance of the entire neural network. The specific neuron agglomerating process of the convolutional layers is shown in Figure 2.

After agglomerating neurons in the fully connected layers and the convolutional layers, we can obtain a compact neural network, which greatly reduces the parameters of the original network. However, the agglomeration of a large number of neurons in the network may cause the loss of accuracy in the network; specifically, the compression ratio is relatively high. We combine the fine-tuning operation to improve the accuracy of the compressed network, and the experiments showed that by fine-tuning the compressed network, we can even get higher accuracy than the original network, as shown in the following section.

**Figure 3:**
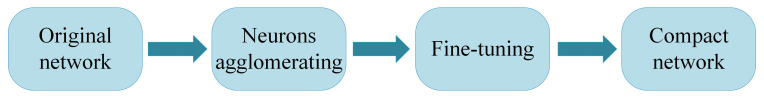
The process of the network compression based on neuron agglomerative clustering.

Based on the above introduction, the overall process of the proposed network compression method based on NAC can be summarized as that shown in Figure 3. First, we fully train the original network, then we apply the NAC algorithm to perform network compression to obtain the compact network. Finally, we fine-tune the compressed network and adjust the parameters of the network. After the above steps, we obtain the final compact network. Here, please note that the proposed network compression method based on NAC can be easily combined with other network compression approaches (e.g., quantization and pruning) to learn more compact networks.

## 4. Experiments and Results

To evaluate the proposed network compression method, we conducted extensive experiments on the Mixed National Institute of Standards and Technology Database (MNIST), CIFAR-10, and CIFAR-100 datasets. Specifically, we applied NAC on three networks: a deep belief network (DBN) and two convolutional neural networks (CNNs). In the following, we report the results of the experiments separately, where the best results shown in the tables are highlighted in boldface.

### 4.1. The Used Datasets

#### 4.1.1. MNIST

MNIST is a commonly used dataset in the computer vision field to test the methods for handwritten digit recognition. The dataset has 10 categories and contains 70,000 gray scale images in total. Among them, 60,000 images are used to train the neural networks, and the other 10,000 images are used for testing. Each image 28 × 28 in size.

#### 4.1.2. CIFAR

The CIFAR-10 dataset contains 60,000 color images in 10 classes, while each class has 6000 images. It is divided into 50,000 training images and 10,000 test images. Each image is 32 × 32 × 3 in size. Specifically, the training data are randomly divided into five training batches, and each batch has 10,000 images. Alternatively, the CIFAR-100 dataset has 100 classes, while each class contains 600 images. In addition, each class has 500 training images and 100 test images. The image size is 32 × 32 × 3.

### 4.2. Results on the MNIST Dataset

**Figure 4:**
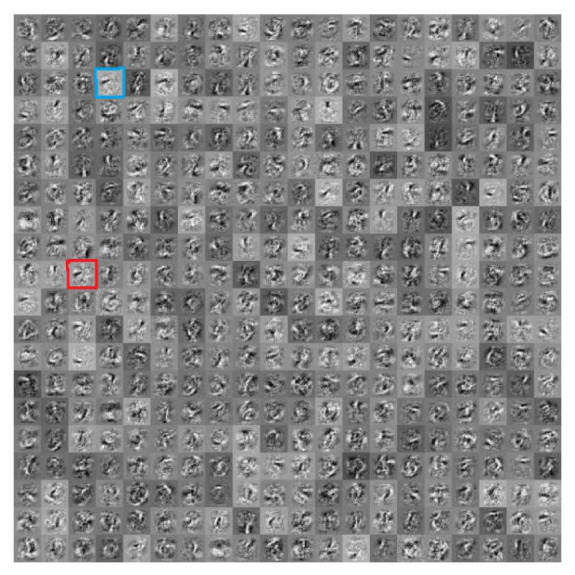
Visualization of neurons in the first hidden layer of the original DBN.

The experiments in this section were performed on the deep belief network (DBN) as presented in Table 1. Figure 4 visualizes the neurons in the first hidden layer of the well-trained DBN. It can be seen from Figure 4 that several neurons in the first hidden layer learn similar features. For instance, the 44th (highlighted with the blue rectangle) and 183rd (highlighted with the red rectangle) neurons almost show the same pattern. This fact indicates that neurons in the original DBNs are severely redundant. Therefore, to obtain a compact network structure, it is necessary to reduce the redundancy of the neurons in it.

In our experiments, we first reduced the neurons in each layer of the original DBN to 300, 300, and 1000, respectively, and obtained a compact network with a compression ratio of 61.68%. The experimental results are shown in Table 1. From Table 1, we can see that even without fine-tuning, our network compression method NAC has almost no loss of accuracy compared to the original network. This proves that the proposed network compression method effectively agglomerates redundant neurons in the network. Furthermore, this also demonstrates the feasibility and rationality of the network compression method proposed in this paper.

To further verify the effectiveness of NAC at a higher compression ratio, we set the number of neurons in the three layers of the DBN to 200, 100, and 100, respectively, for network compression. The classification results are shown in Table 2. When the compression ratio reaches 88.62%, compared with the original network, the compressed network after fine-tuning obtains a higher accuracy than the original network, which justifies the effectiveness of the proposed network compression method in this paper.

In order to investigate whether the good performance of the compressed network can be attributed to the new network architecture and for a more fair comparison, we rebuilt a neural network with the same architecture (the numbers of neurons in the hidden layers are 200, 100, and 100). We fully trained it and fine-tuned it. In the end, the error rate of the reconstructed network is 1.16%, higher than that of the compressed network. Therefore, the good performance of the compressed network cannot be attributed to its new network architecture, but to the reduction of the redundant information in the original network.

Subsequently, we verified the effectiveness of the proposed NAC method on a convolutional neural network (CNN). The structure of the convolutional neural network we used is shown in Table 3. After we fully trained this CNN, we used the proposed NAC method to compress it. The classification results are shown in the third column of Table 3, and as we can see, the compressed network after fine-tuning obtained a higher accuracy than the original network. Comparing the original network and the compressed network, we can see that our network compression method is also quite effective for CNNs. Moreover, this demonstrates the feasibility and versatility of the proposed NAC method again.

### 4.3. Results on the CIFAR Datasets

In the following experiments, we verified the effectiveness of our proposed NAC method on a relatively deeper network, the classic VGGNet-16. To show the superiority of the agglomerative clustering algorithm, we compared it with the *k*-means clustering method [2], which is one of our methods built for network compression.

Table 4 shows the experimental results of compressing VGGNet-16 on the CIFAR-10 and CIFAR-100 datasets. VGGNet (Model-A) corresponds to the results of network compression based on the *k*-means clustering of neurons, while VGGNet (Model-B) corresponds to the network compression based on agglomerative clustering of neurons. From Table 4, we can see that no matter whether *k*-means clustering or agglomerative clustering is used for network compression on the CIFAR-10 dataset, when the compression ratio is as high as 92.96%, our network compression method can still obtain higher accuracy than the original network. This demonstrates that the proposed network compression method is very effective to deep CNNs. By comparing the results of Model-A and Model-B on both the CIFAR-10 and CIFAR-100 datasets, we can see that the results obtained using agglomerative clustering for network compression are consistently better than those obtained using *k*-means clustering. This is because the *k*-means clustering algorithm needs to randomly initialize the cluster centers. This may affect the effect of the *k*-means clustering, e.g., obtaining different cluster centers due to different initializations of the cluster centers, which may further affect the performance of the compressed network. Therefore, the agglomerative clustering is more suitable for the network compression tasks, as it is a much more stable algorithm than *k*-means clustering, basically delivering same positions of the cluster centers given the compression ratio. Overall, the results shown in Table 4 demonstrate the effectiveness of the proposed network compression method, NAC.

Table 5 shows the comparison results of network compression on the CIFAR-10 and CIFAR-100 datasets using agglomerative clustering, *k*-means clustering, and randomly merging the neurons without any clustering method. For a fair comparison, the three methods compressed the same number of neurons in each layer of the original network, and the test errors in the tables were obtained by directly testing the compressed network without fine-tuning. By comparing NAC with the method based on randomly merging neurons, we can see from Table 5 that NAC has almost no harm on the performance of the original neural network, but the method based on randomly merging neurons causes severe damage to the performance of the original network. At the same time, by comparing the results obtained by applying the agglomerative clustering and the *k*-means clustering, it is obviously shown that the results obtained by applying the agglomerative clustering are consistently better than that obtained by applying the *k*-means clustering. This is not because of the influence of fine-tuning, but because the agglomerative clustering algorithm itself is more suitable for the similar neurons’ clustering.

To further demonstrate the effectiveness of the proposed network compression method, we compared NAC with three other state-of-the-art network compression methods. Among them, the work in [63] enforced the channel-level sparsity of CNNs; the method presented in [5] pruned filters from CNNs that were identified as having a small effect on the output accuracy; while [64] categorized all the parameters into two parts at each training iteration and updated them using different rules. The results obtained on the CIFAR-10 and CIFAR-100 datasets are shown in Table 6. For a fair comparison, we used the same network structure provided in [63]. From Table 6, we can see that the model obtained by NAC has higher accuracy when the compression ratio is similar for all the compared methods. This justifies the superiority of the network compression method based on NAC over existing approaches.

## 5. Conclusions

In this paper, we propose a novel neural network compression method based on neuron agglomerative clustering (NAC). Built upon the fact that neurons and connections in the deep neural networks are redundant, we use NAC to agglomerate similar neurons and their corresponding connections. For concreteness, we apply the agglomerative clustering algorithm to cluster neurons in the same layer and find similar neurons in each layer, and then, the similar neurons and their corresponding connections are agglomerated together. Finally, we fine-tune the compressed network to obtain a compact network with no loss of accuracy compared to the original network. We conducted experiments on DBN and two CNNs, including the classic VGGNet and obtained excellent experimental results. In particular, we compared NAC with two state-of-the-art network compression approaches on VGGNet and obtained better classification results than them. These experimental results faithfully demonstrate the effectiveness of the proposed NAC method for network compression.

## Figures and Tables

**Table 1:** The result of the original and compressed DBN on the MNIST dataset.

	Original Network	Compressed Network
1st layer	500	300
2nd layer	500	300
3rd layer	2000	1000
Parameters	1.67 M	0.64 M
Error rate (%)	1.03	1.17
Error rate with fine-tuning (%)	-	0.98

**Table 2:** Architecture and performance of the compressed network with a high compression ratio.

	Original Network	Compressed Network
1st layer	500	200
2nd layer	500	100
3rd layer	2000	100
Parameters	1.67 M	0.19 M
Error rate (%)	1.03	15.8
Error rate with fine-tuning (%)	-	1.01

**Table 3:** The classification results of the original and compressed convolutional neural networks tested on the Mixed National Institute of Standards and Technology Database (MNIST).

	Original Network	Compressed Network
conv_1	6	6
conv_2	6	5
conv_3	16	12
conv_4	16	12
conv_5	120	80
fc_1	120	60
Parameters	0.15 M	0.05 M
Error rate (%)	0.69	5.14
Error rate with fine-tuning (%)	-	0.63

**Table 4:** The results obtained on the CIFAR-10 and CIFAR-100 datasets. P-Pruned refers to the pruned ratio of parameters, and F-Pruned refers to the pruned ratio of floating-point operations per second (FLOPs). The best results are highlighted with bold face.

Datasets	Model	Test Error (%)	Parameters	P-Pruned	FLOPs	F-Pruned	Δ Accuracy (%)
	VGGNet (Baseline)	6.38	33.65 M	-	6.65 $\times \;10^{8}$	-	-
CIFAR-10	VGGNet (Model-A)	6.19	2.37 M	92.96%	3.72 $\times \;10^{8}$	44.06%	+0.19
	VGGNet (Model-B)	**6.08**	2.37 M	**92.96%**	3.72 $\times \;10^{8}$	44.06%	**+0.30**
	VGGNet (Baseline)	26.38	34.02 M	-	6.65 $\times \;10^{8}$	-	-
CIFAR-100	VGGNet (Model-A)	26.30	6.43 M	81.10%	4.93 $\times \;10^{8}$	25.86%	+0.08
	VGGNet (Model-B)	**26.21**	6.43 M	**81.10%**	4.93 $\times \;10^{8}$	25.86%	**+0.17**

**Table 5:** Comparison results between neuron agglomerative clustering (NAC) and network compression by randomly merging neurons and using *k*-means clustering on the CIFAR-10 and CIFAR-100 datasets.

Datasets	Method	Test Error (%)
	Randomly merging neurons	72.38
CIFAR-10	Using *k*-means clustering	6.35
	Using agglomerative clustering	**6.29**
	Randomly merging neurons	87.53
CIFAR-100	Using *k*-means clustering	29.62
	Using agglomerative clustering	**26.93**

**Table 6:** Comparison between NAC and two related approaches on the CIFAR-10 and CIFAR-100 datasets.

Datasets	Method	Parameters Pruned	Δ Accuracy (%)
	Network Slimming [63]	88.5%	+0.14
CIFAR-10	Pruning Filters [5]	88.5%	-0.54
	Global Sparse Momentum [64]	88.5%	+0.20
	Our Method	88.6%	**+0.28**
	Network Slimming [63]	76.0%	+0.22
CIFAR-100	Pruning Filters [5]	75.1%	-1.62
	Global Sparse Momentum [64]	76.5%	+0.08
	Our Method	76.6%	**+0.25**
